# Supplementary material for: Tailoring smart hydrogels through manipulation of heterogeneous subdomains
Source: Nat Commun. 2024 Oct 27;15:9268. doi: 10.1038/s41467-024-53552-3 (PMC11514287; doi:10.1038/s41467-024-53552-3)
Supplement: Supplementary file 1 — Supplementary Information [file 41467_2024_53552_MOESM1_ESM.pdf]

Supplementary Information

Tailoring Smart Hydrogels Through Manipulation of Heterogeneous Subdomains

Haoqing Yang, Tengxiao Liu, Lihua Jin, Yu Huang*, Xiangfeng Duan*, and Hongtao Sun*

Corresponding authors: yhuang@seas.ucla.edu; xduan@chem.ucla.edu; hongtao.sun@psu.edu

This file includes:

Supplementary Note
Supplementary Figures. 1-23
Supplementary Tables 1-7

Other Supplementary Material for this manuscript includes the following:

Supplementary Movies 1-2 (.mp4)

Supplementary Note

Calculation of strain energy density. We assume: (1) all hydrogel film samples maintain the same film thickness, thus the volume fractions (V_i) of various local regions are represented by their corresponding area fractions (A_i); (2) The elastic moduli of homogeneous films obtained from stress-strain curves are used for calculating the local domains in patterned HC-50 and HC-90 samples.

Equations:

In the elastic deformation region: $\sigma = E\varepsilon$,

Strain energy density: $u = \frac{U}{V} = \frac{\sigma^2}{2E} = \frac{E\varepsilon^2}{2} = \frac{\sigma\varepsilon}{2}$

The sum of the weighted strain energy density: $U_i = \sum_1^i (u_i \cdot A_i)\, , i = 1, 2, 3 \ldots$

Known parameters:

$E_{20s} = 35\ kPa$, $E_{40s} = 172\ kPa$, $E_{200s} = 635\ kPa$; *elastic moduli are obtained from the stress-strain curves in Fig. 2a and Supplementary Figs. 1a and 5a.*

Calculations of strain energy density for homogeneous films and patterned hydrogels:

When the soft film or film domain is exposed for 20 seconds and the patterned domain for 200 seconds, the strain energy density is calculated at a stretch of 1.2 (20% global strain):

The strain energy density of the thin film: $u_{film-20s} = \frac{\sigma\varepsilon}{2} = \frac{6\times0.2}{2} = 0.60\, J\ m^{-3}$

The strain energy density of HC-50: $u_{HC-50} = \frac{\sigma\varepsilon}{2} = \frac{46.5\times0.2}{2} = 4.65\, J\ m^{-3}$

The strain energy density of HC-90: $u_{HC-90} = \frac{\sigma\varepsilon}{2} = \frac{43.6\times0.2}{2} = 4.36\, J\ m^{-3}$

When the soft film or film domain is exposed for 40 seconds and the patterned domain for 200 seconds, the strain energy density is calculated at a stretch of 1.2 (20% global strain):

The strain energy density of thin film: $u_{film-40s} = \frac{\sigma\varepsilon}{2} = \frac{32\times0.2}{2} = 3.20\, J\ m^{-3}$

The strain energy density of HC-50: $u_{HC-50} = \frac{\sigma\varepsilon}{2} = \frac{65.2\times0.2}{2} = 6.52\, J\ m^{-3}$

The strain energy density of HC-90: $u_{HC-90} = \frac{\sigma\varepsilon}{2} = \frac{69.7\times0.2}{2} = 6.97\, J\ m^{-3}$

Calculations of strain energy density for local domains:

For HC-50 hydrogels under a global strain of 20%, when the soft film domain is exposed for 20 seconds and the patterned domain for 200 seconds, the estimated local strain profiles were obtained from strain mapping. The defined local regions in HC-50 cellular unit are shown in Supplementary Fig. 10a.

Subdomain A: $A_A = 19.4\%$

We assume the y strain distributions among the subdomains A and B are $\varepsilon_A(x,y)$ and $\varepsilon_B(x,y)$. According to the y-strain mapping and linear profiles across various domains, we can obtain the estimated y strain profile of the subdomain A within the x-y plane:

$$\varepsilon_A(x,y) = 0.12cos(4.48x) \cdot y + 0.45y + 0.12cos(2.41y) \cdot x + 0.45x + 0.57$$

Integrating $\varepsilon_A(x,y)$ over the subdomain A region, where $x \approx (-0.35, +0.35)$, $y \approx (-0.65, +0.65)$:

$u_A = \int \frac{E\varepsilon^2}{2} d\varepsilon = \frac{35}{2} \int_{-0.65}^{0.65} \int_{-0.35}^{0.35} (0.12cos(4.48x) \cdot y + 0.45y + 0.12cos(2.41y) \cdot x + 0.45x + 0.57)^2 dxdy \approx 5.98 \, J \, m^{-3}$

Subdomain B: $A_B = 40.9\%$ (the sum of two portions)

The estimated y strain profile of the subdomain B within the x-y plane:

$$\varepsilon_B(x,y) = 0.24cos(4.76x) \cdot y + 0.17y + 0.24cos(2.09y) \cdot x + 0.17x + 0.41$$

Integrating $\varepsilon_B(x,y)$ over the subdomain B region, where $x \approx (-0.33, +0.33)$, $y \approx (-0.75, +0.75)$

$u_B = \int \frac{E\varepsilon^2}{2} d\varepsilon = \frac{35}{2} \int_{-0.75}^{0.75} \int_{-0.33}^{0.33} (0.24cos(4.76x) \cdot y + 0.17y + 0.24cos(2.09y) \cdot x + 0.17x + 0.41)^2 dxdy \approx 3.34 \, J \, m^{-3}$

Inclined region: $A_{Inclined} = 10.4\%$, average $\varepsilon_{Inclined} \approx 2\%$.

$$u_{Inclined} = \frac{E\varepsilon^2}{2} = 0.13 \, J \, m^{-3}$$

Vertical region: $A_{Vertical} = 29.3\%$, average $\varepsilon_{Vertical} \approx 11\%$.

$u_{Vertical} = \frac{E\varepsilon^2}{2} = 3.84 \, J \, m^{-3}$

The sum of the weighted strain energy density: $U_{HC-50} = \sum_1^4 (u_i \cdot A_i) = 3.67 \, J \, m^{-3}$

For HC-90 hydrogels under a global strain of 20%, when the soft film domain is exposed for 20 seconds and the patterned domain for 200 seconds, the estimated local strain profiles were obtained from strain mapping. The defined local regions in HC-90 cellular unit are shown in Supplementary Fig. 10b.

Subdomain A: $A_A = 27.9\%$

We assume the y strain distributions among the subdomains A and B are $\varepsilon_A(x,y)$ and $\varepsilon_B(x,y)$. According to the y-strain mapping and linear profiles across various domains, we can obtain the estimated y strain profile of the subdomain A within the x-y plane:

$$\varepsilon_A(x,y) = 0.06cos(3.49x) \cdot y + 0.22y - 0.14cos(1.74y) \cdot x + 0.42x + 0.28$$

Integrating $\varepsilon_A(x,y)$ over the subdomain A region, where $x \approx (-0.45, +0.45)$, $y \approx (-0.90, +0.90)$

$u_A = \int \frac{E\varepsilon^2}{2} d\varepsilon = \frac{35}{2} \int_{-0.9}^{0.9} \int_{-0.45}^{0.45} (0.06cos(3.49x) \cdot y + 0.22y - 0.14cos(1.74y) \cdot x + 0.42x + 0.28)^2 dxdy \approx 2.95\, J\, m^{-3}$

Subdomain B: $A_B = 37.5\%$ (the sum of two portions)

The estimated y strain profile of the subdomain B within the x-y plane:

$$\varepsilon_B(x,y) = 0.08cos(3.93x) \cdot y + 0.13y - 0.04\cos(1.85y) \cdot x + 0.25y + 0.21$$

Integrating $\varepsilon_B(x,y)$ over the subdomain B region, where $x \approx (-0.40, +0.40)$, $y \approx (-0.85, +0.85)$

$u_B = \int \frac{E\varepsilon^2}{2} d\varepsilon = \frac{35}{2} \int_{-0.85}^{0.85} \int_{-0.4}^{0.4} (0.08cos(3.93x) \cdot y + 0.13y - 0.04\cos(1.85y) \cdot x + 0.25x + 0.21)^2 dxdy \approx 1.97\, J\, m^{-3}$

Inclined region: $A_{Inclined} = 22.7\%$, average $\varepsilon_{Inclined} \approx 3\%$.

$u_{Inclined} = \frac{E\varepsilon^2}{2} = 0.286\, J\, m^{-3}$

Vertical region: $A_{Vertical} = 11.9\%$, average $\varepsilon_{Vertical} \approx 10\%$.

$u_{Vertical} = \frac{E\varepsilon^2}{2} = 3.175\, J\, m^{-3}$

The sum of the weighted strain energy density: $U_{HC-90} = \sum_1^4 (u_i \cdot A_i) = 2.00\, J\, m^{-3}$

For HC-50 hydrogels under a global strain of 20%, when the soft film domain is exposed for 40 seconds and the patterned domain for 200 seconds, the estimated local strain profiles were obtained from strain mapping. Notably, as UV exposure increased in the soft film regions from 20 to 40 seconds, it indicates a reduced discernibility of the strain-induced heterogeneities within the soft domains (Supplementary Fig. 5c,d).

Subdomain A: $A_A = 22.4\%$, average $\varepsilon_A \approx 21\%$.

$u_A = \frac{E\varepsilon^2}{2} = 3.79\, J\, m^{-3}$

Subdomain B: $A_B = 46.8\%$, average $\varepsilon_B \approx 18\%$.

$u_B = \frac{E\varepsilon^2}{2} = 2.79\, J\, m^{-3}$

Inclined region: $A_{Inclined} = 14.8\%$, average $\varepsilon_{Inclined} \approx 5\%$.

$u_{Inclined} = \frac{E\varepsilon^2}{2} = 0.79\, J\, m^{-3}$

Vertical region: $A_{Vertical} = 16.0\%$, average $\varepsilon_{Vertical} \approx 14\%$.

$u_{Vertical} = \frac{E\varepsilon^2}{2} = 6.22\, J\, m^{-3}$

The sum of the weighted strain energy density: $U_{HC-50} = \sum_1^4 (u_i \cdot A_i) = 3.27\, J\, m^{-3}$

For HC-90 hydrogels under a global strain of 20%, when the soft film domain is exposed for 40 seconds and the patterned domain for 200 seconds, the estimated local strain profiles were obtained from strain mapping.

Subdomain A: $A_A = 22.4\%$, average $\varepsilon_A \approx 19\%$.

$u_A = \frac{E\varepsilon^2}{2} = 3.11\, J\, m^{-3}$

Subdomain B: $A_B = 46.1\%$, average $\varepsilon_B \approx 12\%$.

$u_B = \frac{E\varepsilon^2}{2} = 1.24\, J\, m^{-3}$

Inclined region: $A_{Inclined} = 15.0\%$, average $\varepsilon_{Inclined} \approx 10\%$.

$u_{Inclined} = \frac{E\varepsilon^2}{2} = 3.18\, J\, m^{-3}$

Vertical region: $A_{Vertical} = 16.5\%$, average $\varepsilon_{Vertical} \approx 12\%$.

$u_{Vertical} = \frac{E\varepsilon^2}{2} = 4.57\, J\, m^{-3}$

The sum of the weighted strain energy density: $U_{HC-90} = \sum_1^4 (u_i \cdot A_i) = 2.50\, J\, m^{-3}$

Notes:

To simplify the calculations of strain energy density (u_i), we made several assumptions that may lead to discrepancies from the actual case. For example, we used the elastic moduli of homogeneous hydrogel films (cured for 20, 40, and 200 seconds) to represent the elastic moduli in the local patterned (200 seconds) and film domains (20 or 40 seconds) when calculating the strain energy density of the patterned hydrogels. In our material system, resistance to deformation in the patterned samples may vary across different regions within the patterned or film domains, leading to variations in elastic moduli across local regions. We also estimated the strain profiles within the x-y plane (using average strain values for $u_{inclined}$ and $u_{vertical}$; or by integrating simplified strain profiles for u_A and u_B) to calculate the strain energy density in localized domains. As a result, the calculated sum of the weighted strain energy densities across local domains ($U_i = \sum_1^i u_i \cdot A_i, i = 1, 2, 3 \ldots$) based on volume/area fractions and local strain energy densities was lower than the strain energy density directly derived from the stress-strain curves of the overall patterned hydrogel samples (HC-50 and HC-90). Additionally, all the calculated parameters are summarized in Supplementary Tables 3-6.

Supplementary Figures

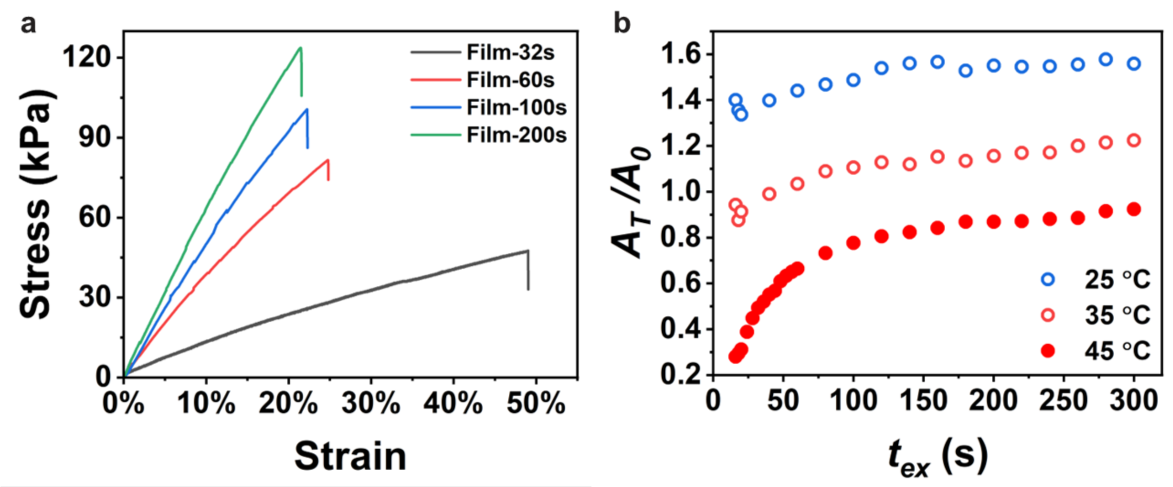

Supplementary Fig. 1. Characteristics of homogeneous hydrogel films without cellular patterned domains. a, The stress-strain curves of synthetic hydrogel films cured under different UV exposure times from 32 to 200 seconds. **b,** A broad range of thermo-responsive shrinking ratios of hydrogel films when varying the UV exposure times from 20 to 300 seconds. A_T is the measured area at responsive temperature T (*e.g.*, shrinkage state: $A_{45\,°C}$, T = 45°C), A_0 is the area of as-printed hydrogel film.

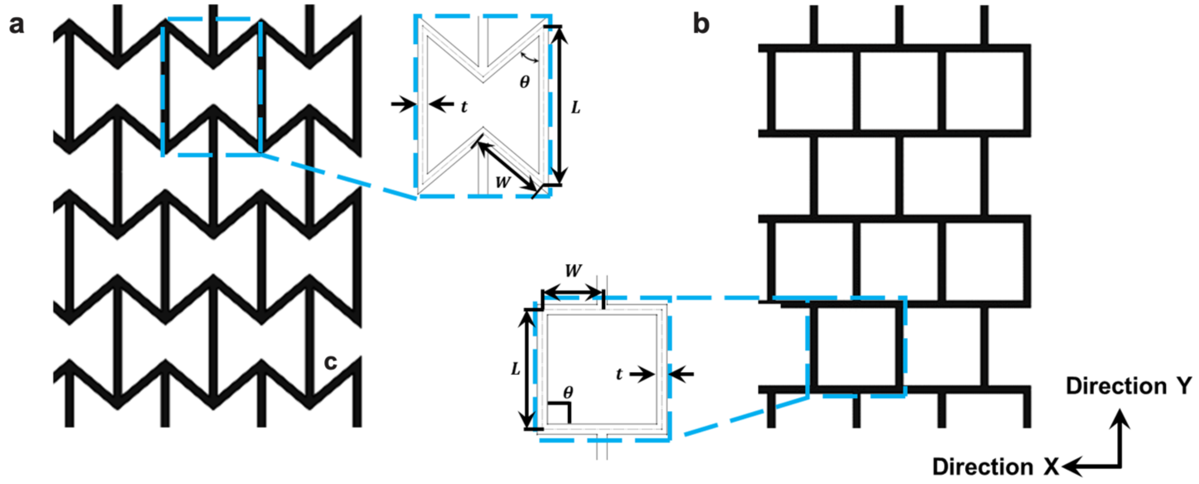

Supplementary Fig. 2. Honeycomb (HC)-structured frameworks with specifically designed angles and Poisson's ratios. a, A re-entrant honeycomb (HC) metamaterial with a specified angle θ of 50° (designated as HC-50). **b,** A honeycomb network with an angle θ of 90° (denoted as HC-90**)**. Basic geometry parameters of these structures: the length of the vertical wall L, the length of the inclined wall W, the wall thickness t, and the inclined angle θ. The Poisson's ratios ν of the HC frameworks without hydrogel films were determined by the equation $\nu_y = -\frac{\epsilon_x}{\epsilon_y} = \frac{(L/W+sin(\theta-90))\times sin(\theta-90)}{cos^2(\theta-90)}$, $\theta = (0,90)$ & $(90,180)$. The various printed wall thicknesses $t = n \cdot r, n = 1,2,3,....$ [28-30].

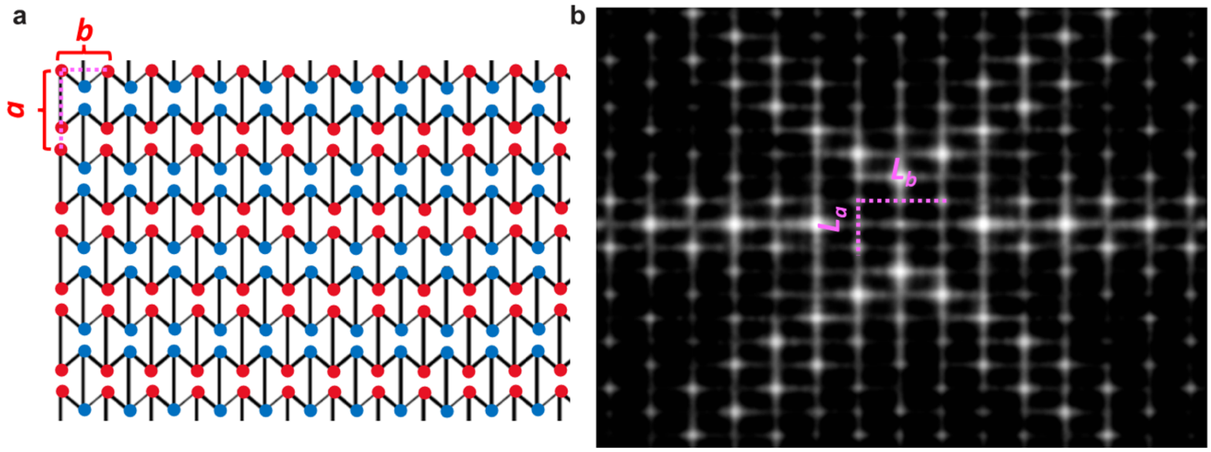

Supplementary Fig. 3. Defined basic parameters. a, Periodic dimension parameters defined in real space. **b,** Corresponding parameters in reciprocal space.

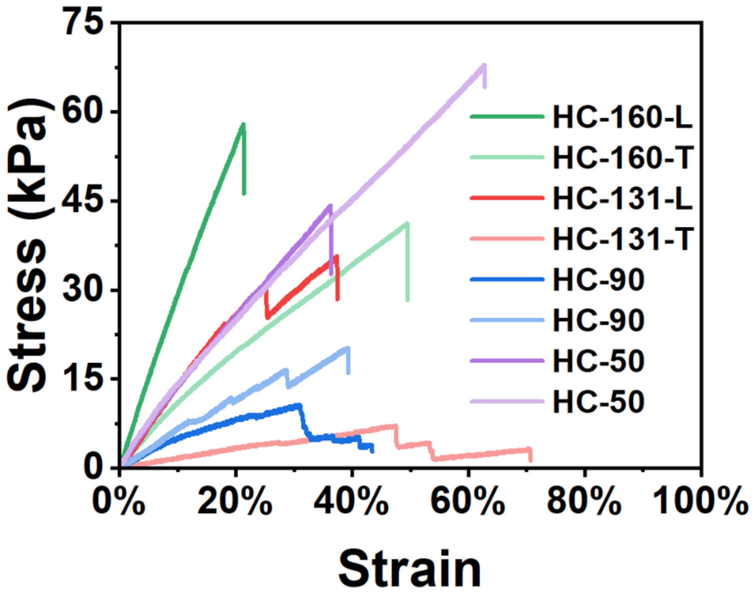

Supplementary Fig. 4. Anisotropic mechanical behaviors of synthetic hydrogels embedded with various HC-patterned domains, when stretched along the x (light-colored line) and y (dark-colored line) directions.

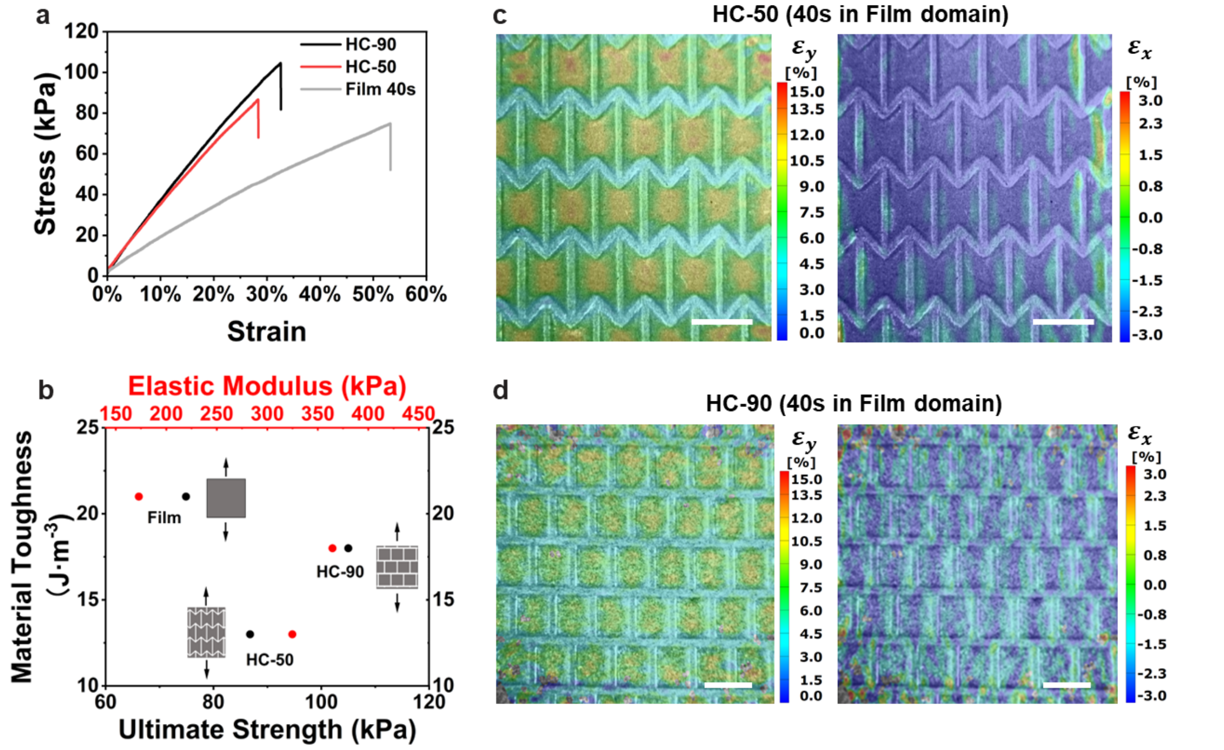

Supplementary Fig. 5. Mechanical characteristics of HC-structured hydrogels with an increased UV curing time in the film domain. a, Stress-strain curves of synthetic hydrogel films embedded with various cellular patterns, such as HC-50 (θ=50°) and HC-90 (θ=90°), along with the homogeneous film. UV curing times are set to 200 seconds in cellular patterned domains and 40 seconds in the film domains and the control homogeneous film. **b,** Correlations between material toughness and strength/elastic modulus, which exhibit reduced damage tolerance. **c,d,** Full-field strain mapping via Digital Imaging Correlation (DIC) analysis for ε_y and ε_x when stretching HC-50 (**c**) and HC-90 (**d**) along the y direction. Imaging of strain mapping is superimposed on the optical imaging of the stretched sample. The heterogeneous subdomains become less discernible due to the reduced contrast in elastic modulus between the cellular patterned domains and the film domains. Scale bar: 2mm.

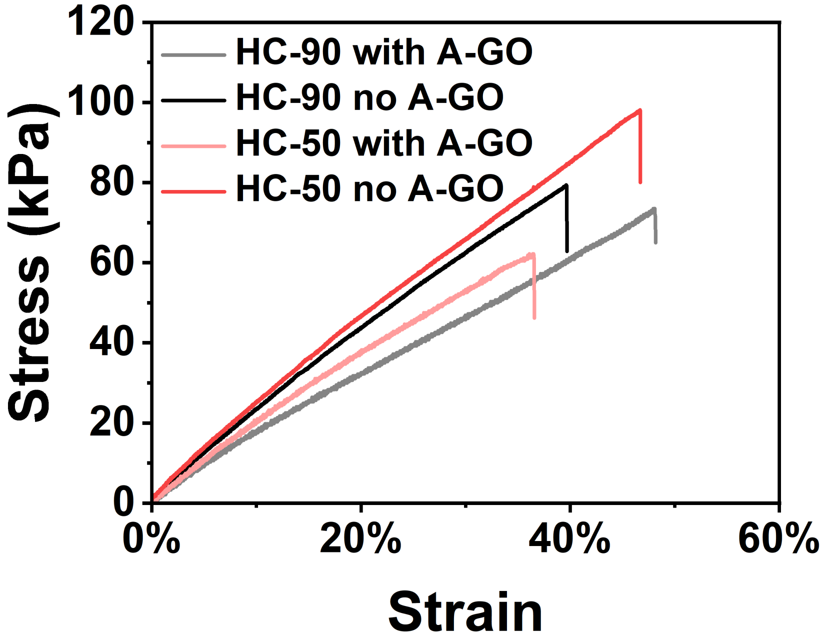

Supplementary Fig. 6. The comparison of stress-strain curves for HC-90 and HC-50 hydrogel samples with and without amine functionalized graphene oxide (A-GO) additives. A low concentration (0.6 mg mL^{-1}) A-GO was prepared in our UV curable resin, acting as tracking markers for DIC analysis. Hydrogels incorporating A-GO exhibited minor variations in mechanical behaviors compared to those printed without A-GO additives. This difference is likely due to the absorption of UV light by A-GO during the printing process.

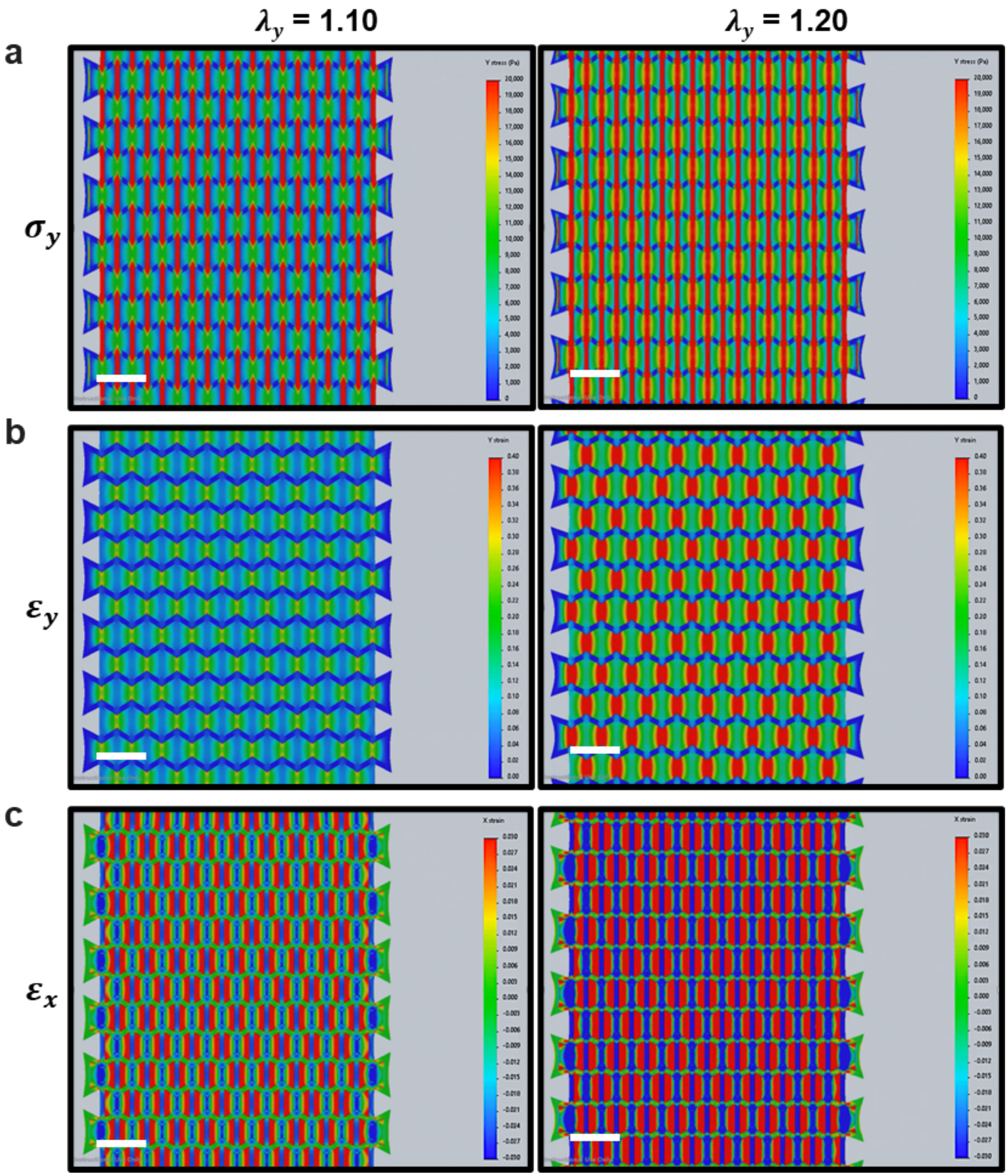

Supplementary Fig. 7. Finite element analysis for σ_y (**a**), ε_y (**b**), and ε_x (**c**) when stretching the HC-50 hydrogel film ($\theta = 50°$) along the y direction. UV curing times are set to 200 seconds in cellular patterned domains and 20 seconds in the film domains within cellular units. Scale bar, 3 mm.

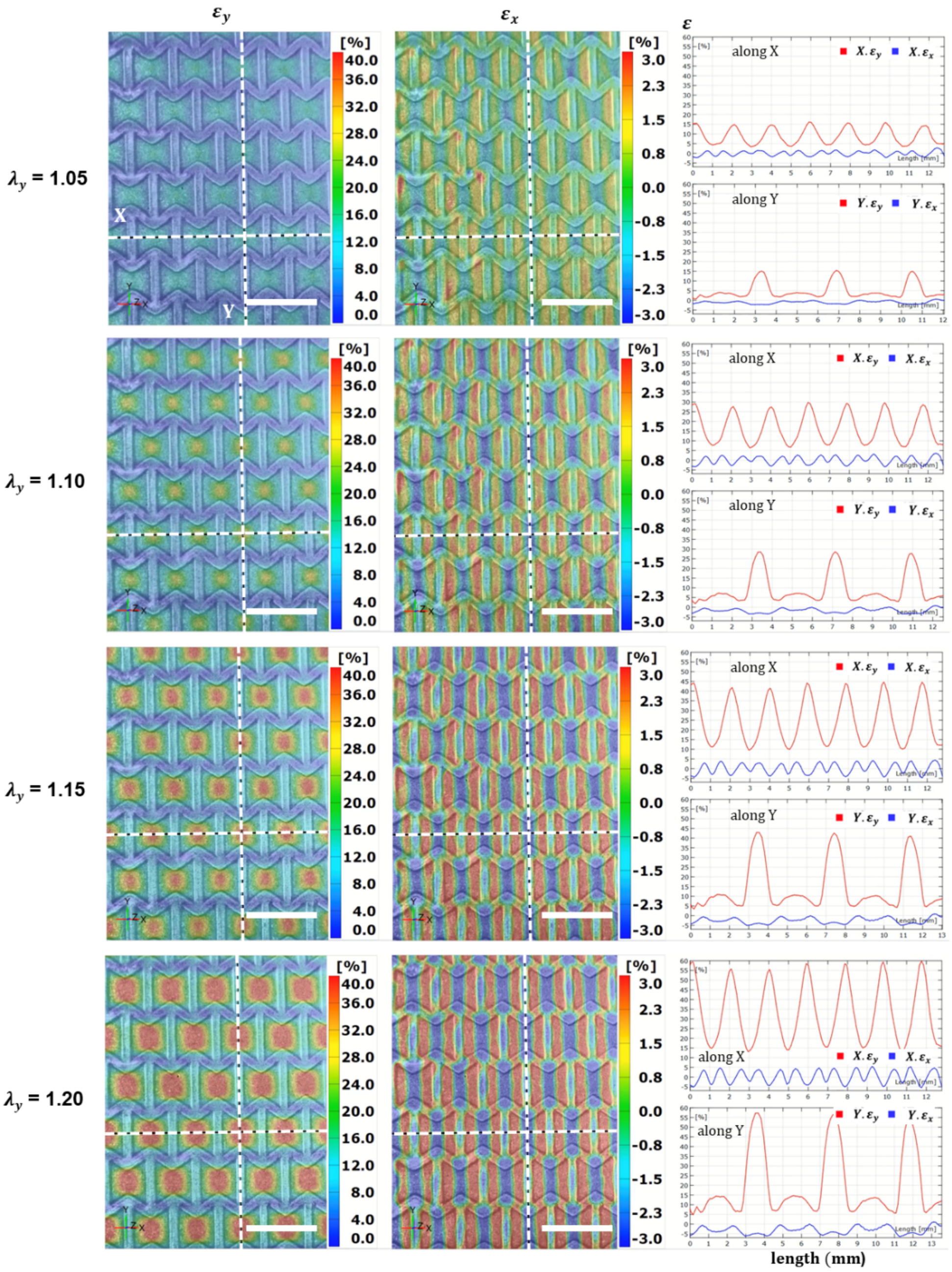

Supplementary Fig. 8. Time-resolved full-field strain mappings via DIC analysis for ε_y and ε_x when stretching the HC-50 hydrogel film along the y direction. Selective x- and y-direction strain profiles along two orthogonal linear pathways (marked as "X" and "Y") are analyzed to determine periodical arrangements of heterogeneous subdomains. The full-field strain mapping results are displayed as superimposed images, integrating optical grayscale images of the hydrogel samples with strain maps to simultaneously capture both structure and deformation information. Scale bar, 3 mm.

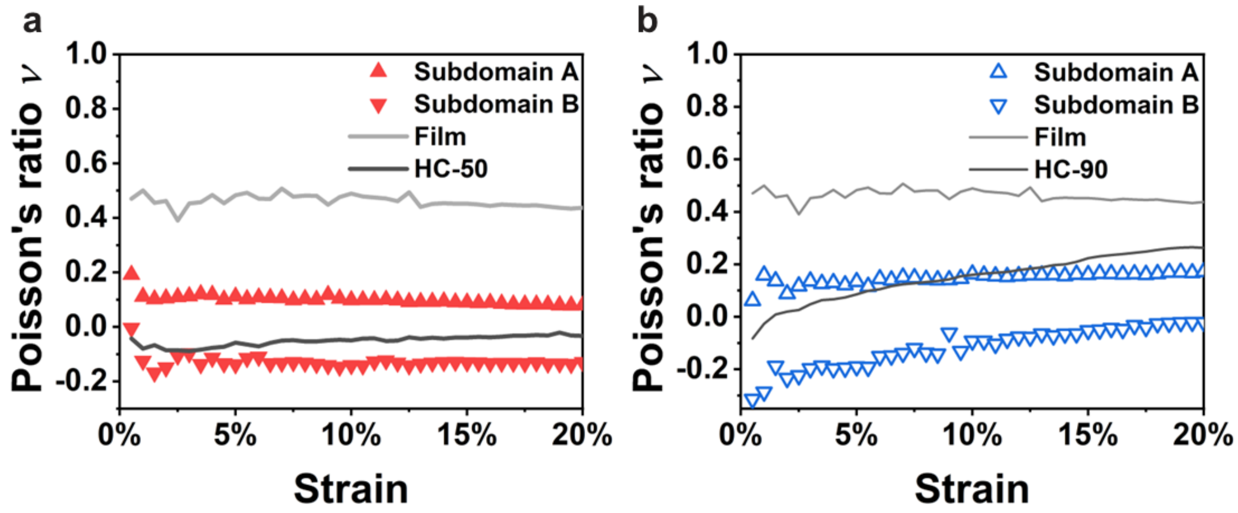

Supplementary Fig. 9. The comparison of Poisson's ratios in local subdomains and overall hydrogel films. a, The HC-50 hydrogel film exhibited substantial differences in Poisson's ratios for local subdomains A and B, differing from those of the overall HC-50 hydrogel and the control homogeneous film. **b,** The HC-90 hydrogel film exhibited differences in Poisson's ratios for local subdomains A and B, and those of the overall HC-structured hydrogels and the control film. The Poisson's ratios were determined from the full-field DIC strain mapping.

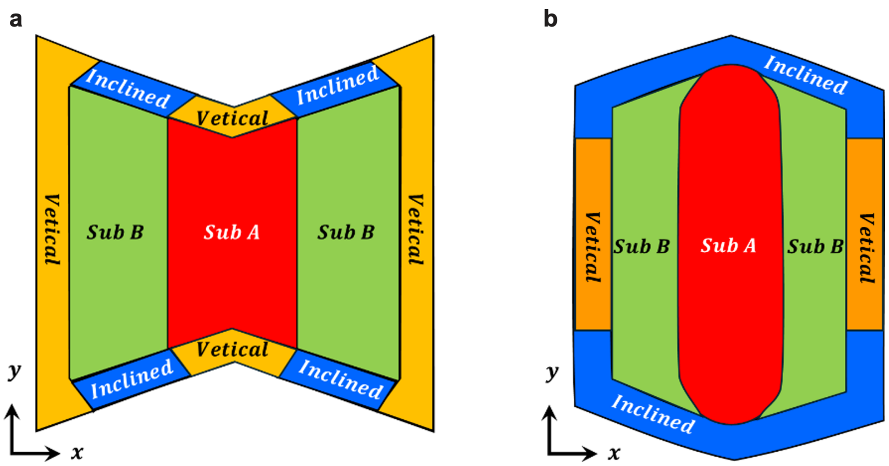

Supplementary Fig. 10. Defined local regions in HC-50 (a) and HC-90 (b) cellular units at 20% strain.

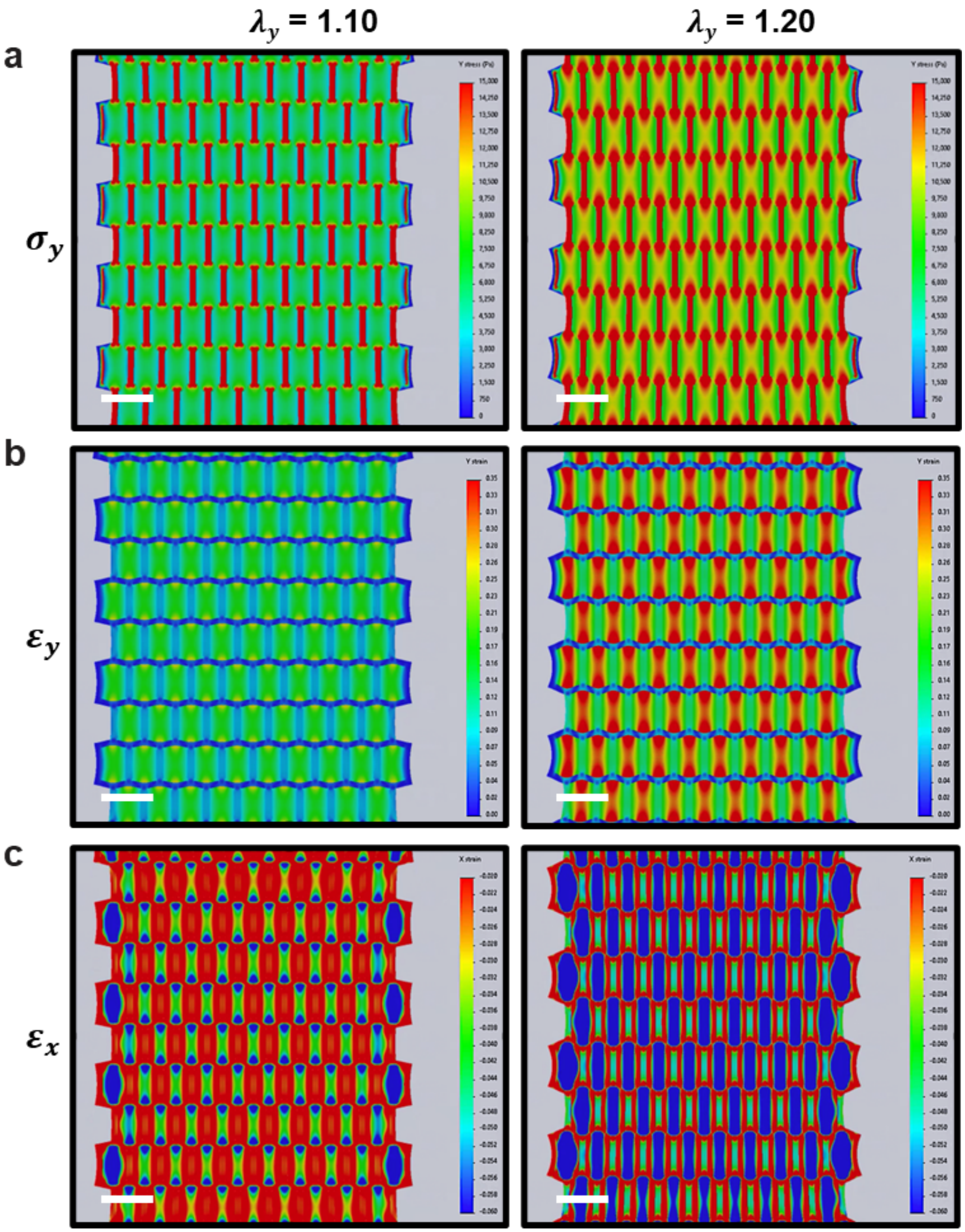

Supplementary Fig. 11. Finite element analysis for σ_y (**a**), ε_y (**b**), and ε_x (**c**) when stretching the HC-90 hydrogel film ($\theta = 90°$) along the y direction. UV curing times are set to 200 seconds in cellular patterned domains and 20 seconds in the film domains within cellular units. Scale bar, 3 mm.

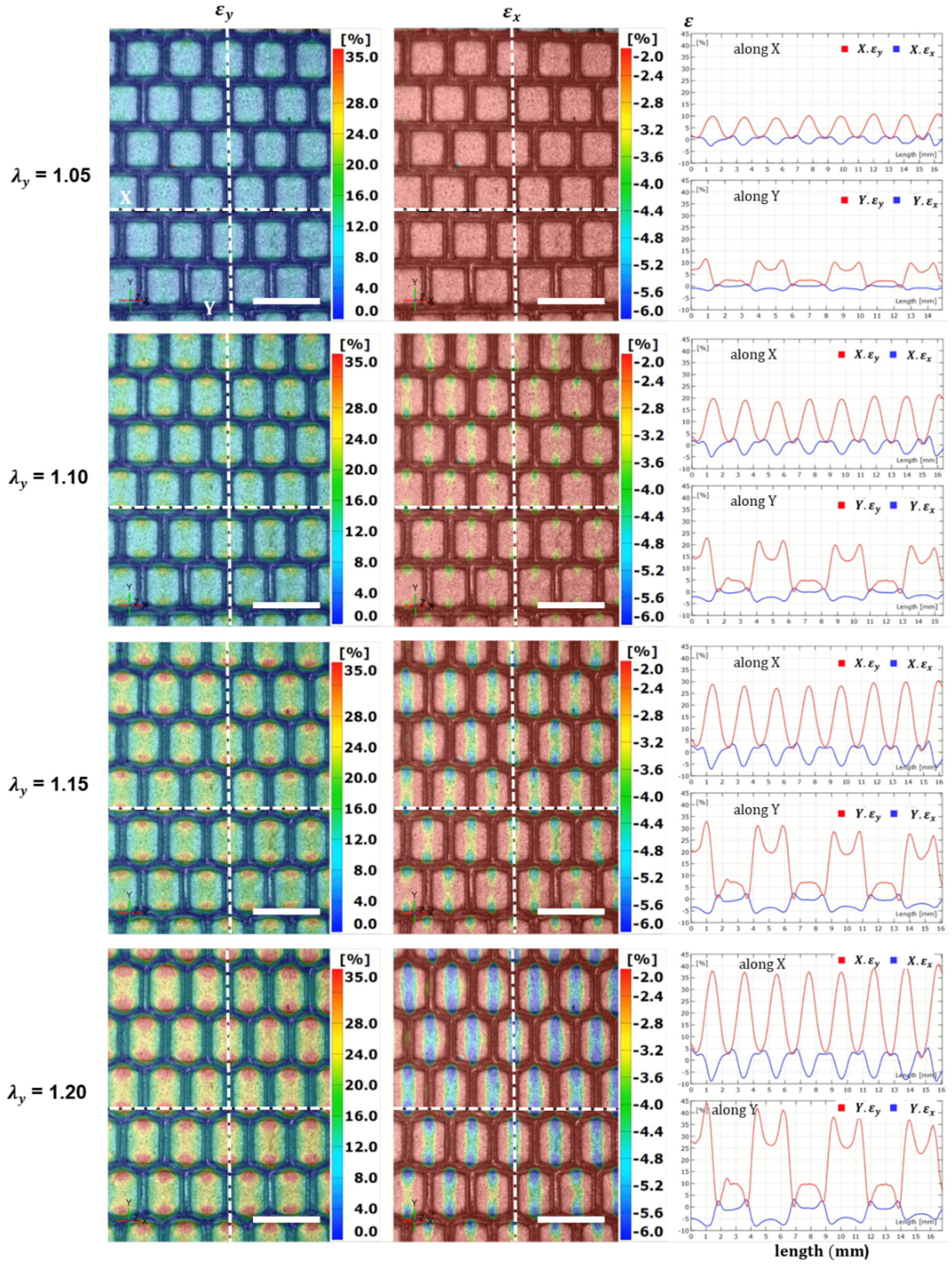

Supplementary Fig. 12. Time-resolved full-field strain mappings via DIC analysis for ε_y and ε_x when stretching the HC-90 hydrogel film along the y direction. Selective x- and y-direction strain profiles along two orthogonal linear pathways (marked as “X” and “Y”) are analyzed to determine periodical arrangements of heterogeneous subdomains. The full-field strain mapping results are displayed as superimposed images, integrating optical grayscale images of the hydrogel samples with strain maps to simultaneously capture both structure and deformation information. Scale bar, 3 mm.

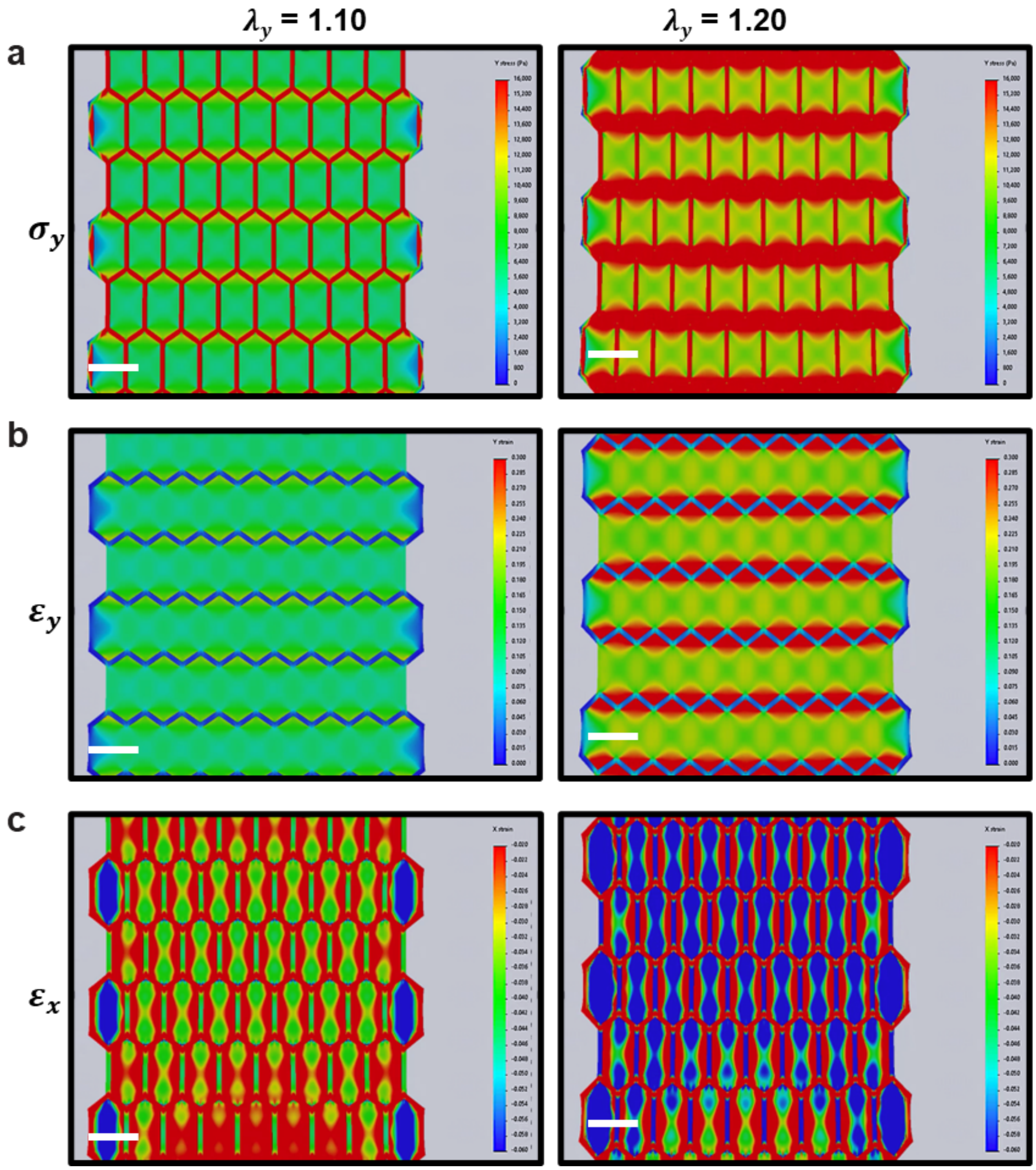

Supplementary Fig. 13. Finite element analysis for σ_y (**a**), ε_y (**b**), and ε_x (**c**) when stretching the HC-120 hydrogel film ($\theta = 120°$) along the y direction. UV curing times are set to 200 seconds in cellular patterned domains and 20 seconds in the domains within cellular units. Scale bar, 3 mm.

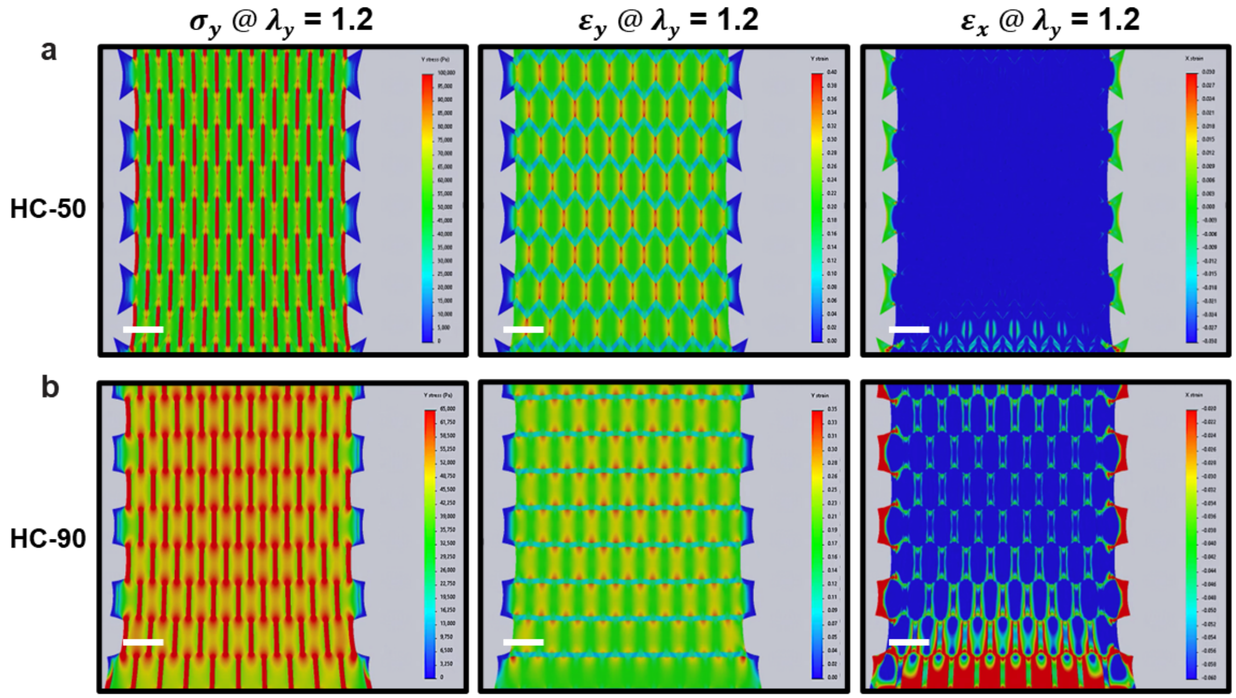

Supplementary Fig. 14. Finite element analysis for HC-50 (**a**) and HC-90 (**b**) hydrogel films at a stretch of 1.2. UV curing times are set to 200 seconds in the cellular patterned domains and 40 seconds in the film domains. Scale bar, 3 mm.

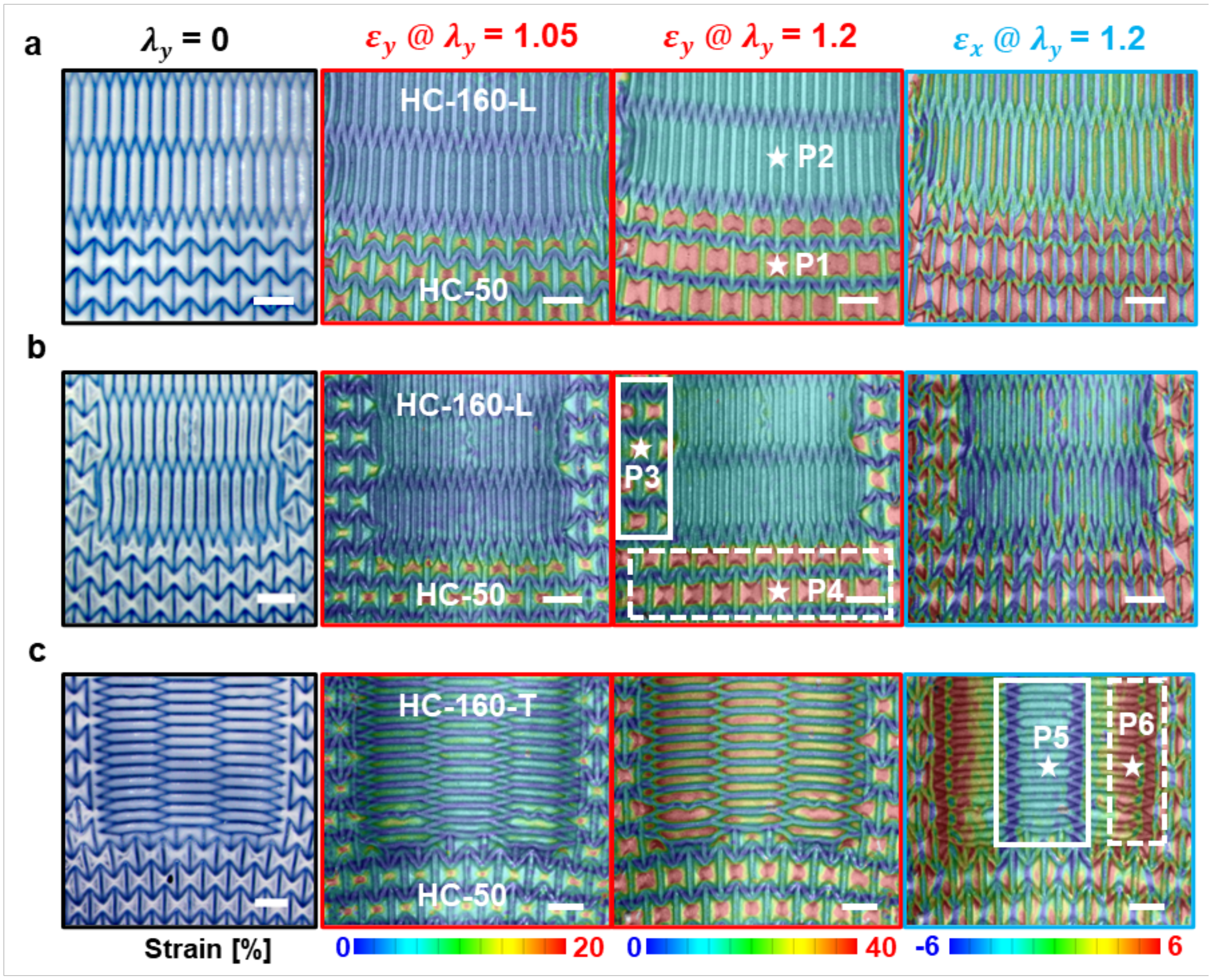

Supplementary Fig. 15. Mechanical characterizations of synthetic hydrogel material system with various designs. a, Illustration of "multi-phase" structure as depicted in HC-160-L&HC-50 hydrogels through full-field strain mapping in real space, with stretching applied along the y-direction. The UV curing times are set at 200 seconds in cellular patterns and 20 seconds in the film domain. **b,c**, Incorporation of inclusion "phases" into synthetic hydrogels, including HC-160-L@HC-50 (**b**) and HC-160-T@HC-50 (**c**) during stretching along the y-direction. Imaging of strain mapping is superimposed on the optical imaging of the stretched sample. The full-field strain mapping results are displayed as superimposed images, integrating optical grayscale images of the hydrogel samples with strain maps to simultaneously capture both structure and deformation information. P1, P3, and P4 refer to the subdomain A of the "HC-50 phase", P2 refers to film domain of "HC-160-L phase", P5 and P6 refer to the central region and near the interface within the "HC-160-T inclusion phase". Scale bars, 2 mm.

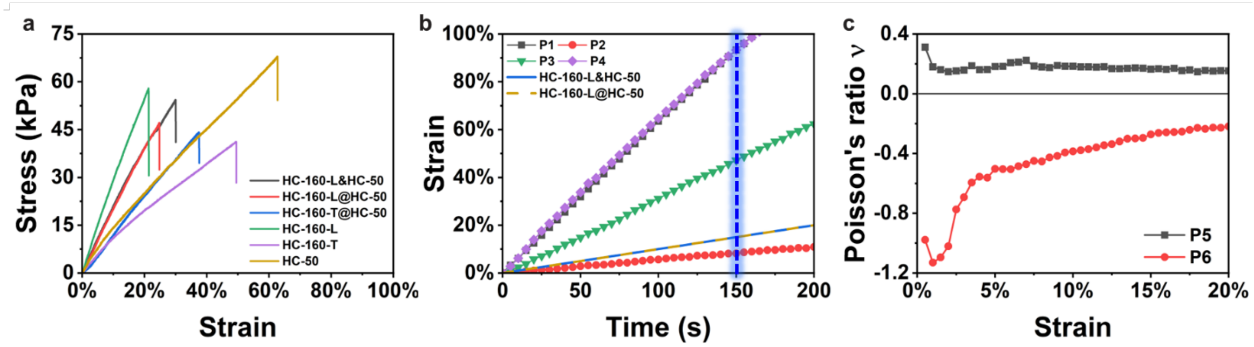

Supplementary Fig. 16. Mechanical characterizations of various anisotropic single "phases" and integrated multiple "phases". a, Stress-strain curves for hydrogel films incorporating distinct "phases", mimicking different microstructures in crystalline materials. **b,** Localized time-dependent strains for selected regions (*e.g.*, P1-P4) derived from sequential time-resolved full-field strain mapping during stretching. **c,** Comparison of distinct Poisson ratios between the central region and near interfacial region of the "HC-160-T inclusion phase" for HC-160-T@HC-50 hydrogel.

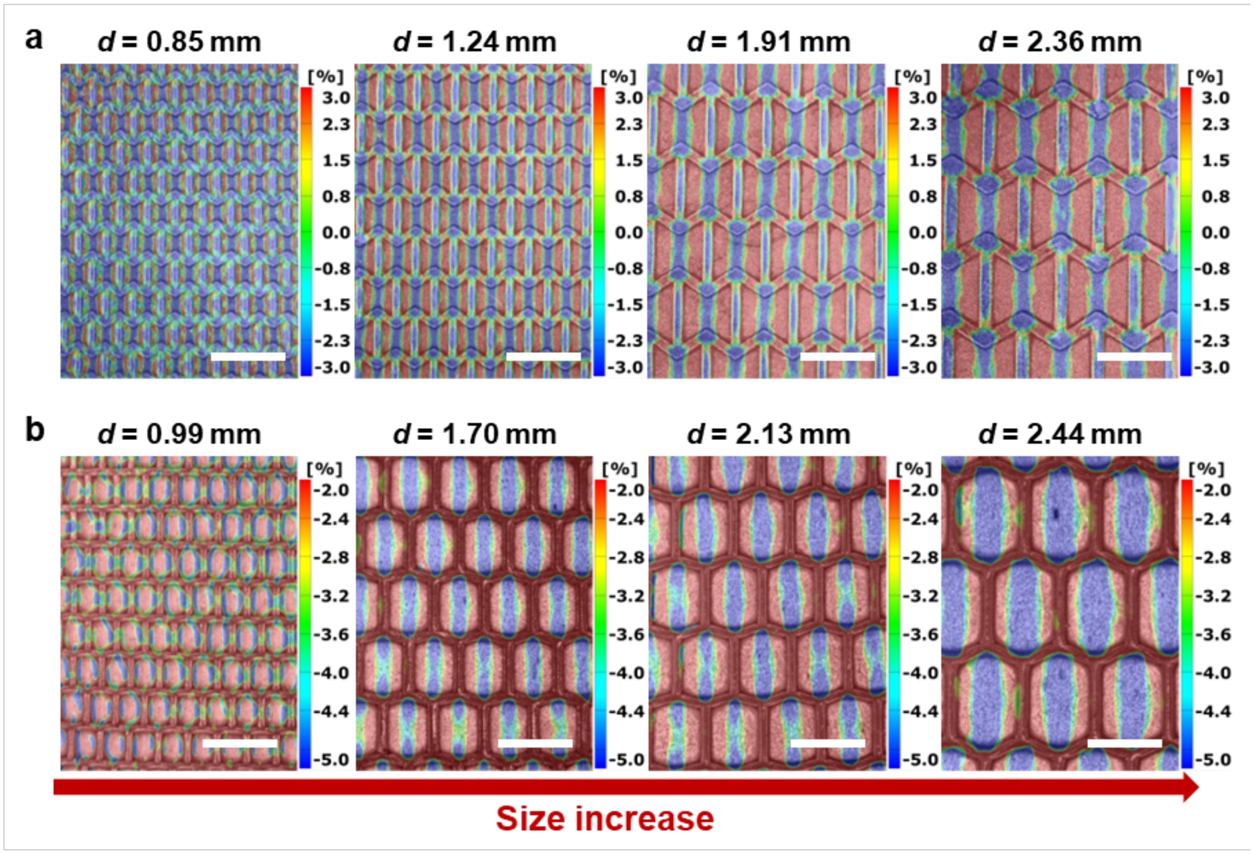

Supplementary Fig. 17. Size-dependent tailoring of heterogeneous subdomains. Full-field x-direction strain mappings illustrate the size-dependent localizations of heterogeneous subdomains in HC-50 (**a**) and HC-90 hydrogels (**b**) under a y-direction stretch (λ_y = 1.2). The UV curing times are set at 200 seconds for the cellular patterned domains and 20 seconds for the film domains. The full-field strain mapping results are displayed as superimposed images, integrating optical grayscale images of the hydrogel samples with strain maps to simultaneously capture both structure and deformation information. The dimension of hydrogel films is 36 mm × 25 mm. Scale bars, 3 mm.

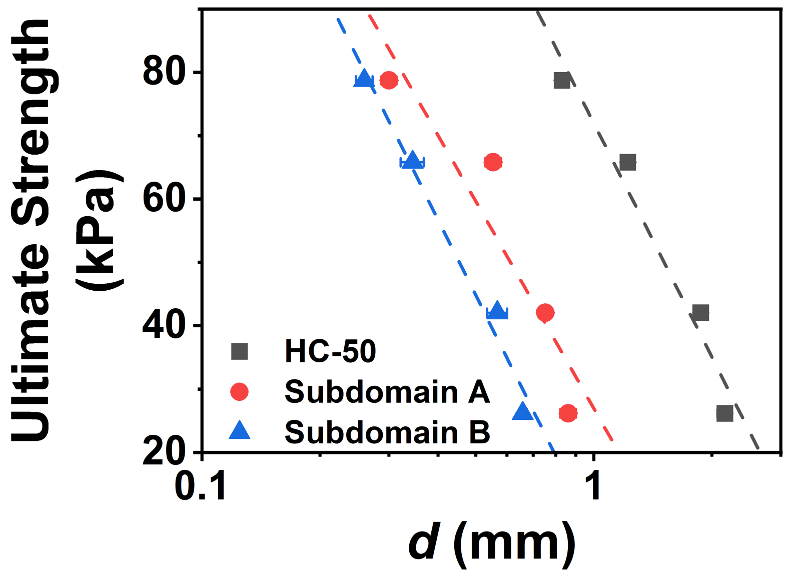

Supplementary Fig. 18. Established correlations between the ultimate strength and the critical dimensions (*e.g.*, d, d_A, and d_B) across a series of HC-50 hydrogel films with varying dimensions. Data were presented as mean values ± SD. The number of replicates n = 3.

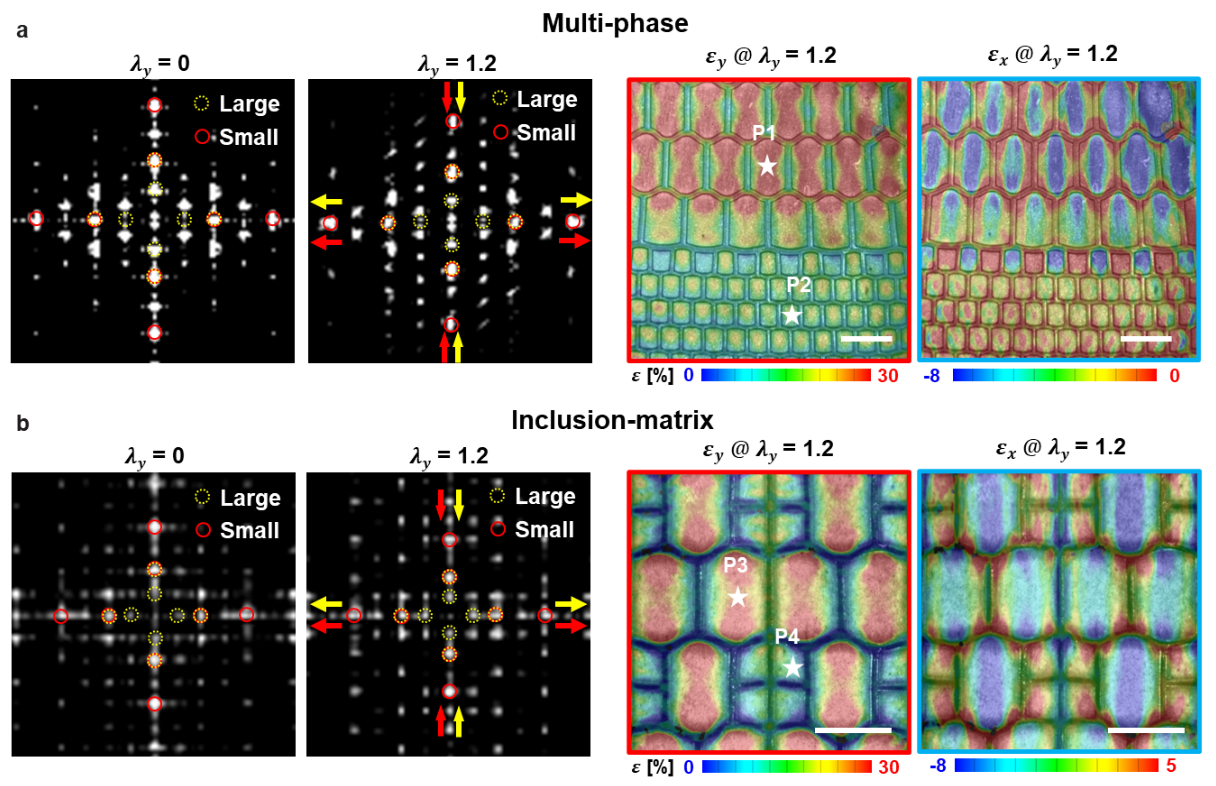

Supplementary Fig. 19. Characterizations of integrated "phases" with differently sized cellular patterns within a single hydrogel film. FFT patterns and strain mappings of integrated hydrogel films under uniaxial stretching along the y-direction, depicting the "multi-phase" (**a**) and "inclusion-matrix" (**b**) configurations. Under stretch, the two distinct phases, composed of large and small cellular units in real space, are evident in the corresponding FFT patterns, marked by yellow dashed circles and red solid circles, respectively, in reciprocal space. Full-field strain mapping results are displayed as superimposed images, integrating optical grayscale images of the hydrogel samples with strain maps to simultaneously capture both structure and deformation information. Scale bars, 3 mm.

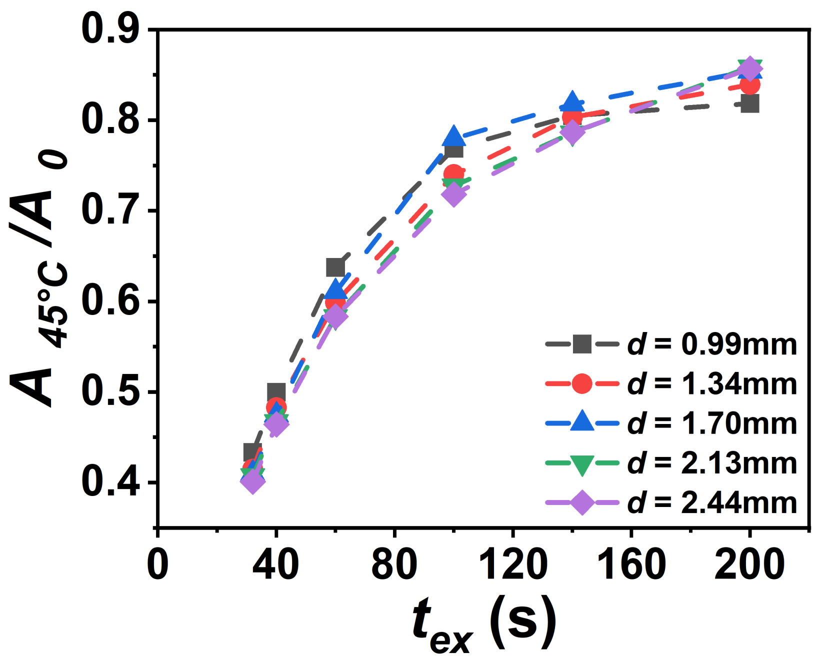

Supplementary Fig. 20. Global shrinking ratio ($A_{45°C}/A_0$) versus local exposure time (t_{ex}) at the cellular patterned domains in HC-90 hydrogel films with varying dimensions of cellular units (d = 0.99-2.44 mm). The local UV curing times at the cellular patterned domains vary from 40 to 200 seconds, with a fixed curing time of 20 seconds in the film domain.

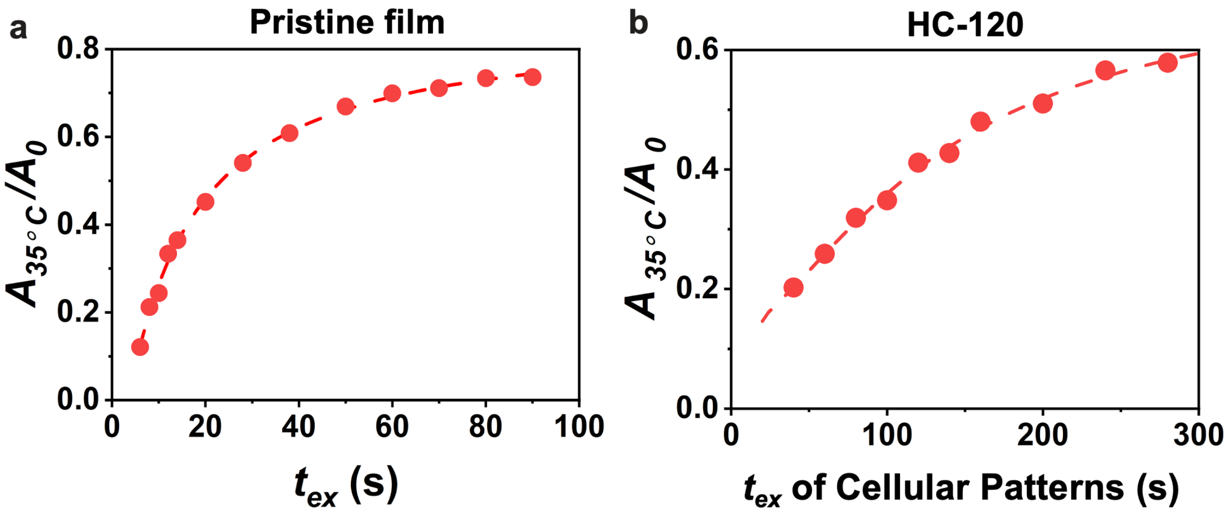

Supplementary Fig. 21. Master curves of curing time-dependent shrinking ratios for PNIPAm hydrogel films under physiological conditions (*e.g.*, 35 °C). a, Shrinking ratio ($A_{35°C}/A_0$) versus exposure time (t_{ex}) in homogeneous hydrogel films. The UV curing time varies from 6 to 96 seconds in the film, resulting in a shrinking ratio ranging from 0.12 to 0.74. **b,** Shrinking ratio ($A_{35°C}/A_0$) versus exposure time (t_{ex}) for HC-120 hydrogels when varying the curing time at the local cellular pattern domains from 40 to 280 seconds (maintaining curing time of 20 seconds at the film domain), leading to a wide shrinking ratio range of 0.20-0.58. NIPAm monomer instead of co-monomers was chosen to lower the T_c of our hydrogel, enabling shape transformations near physiological temperatures (*e.g.*, 35 °C).

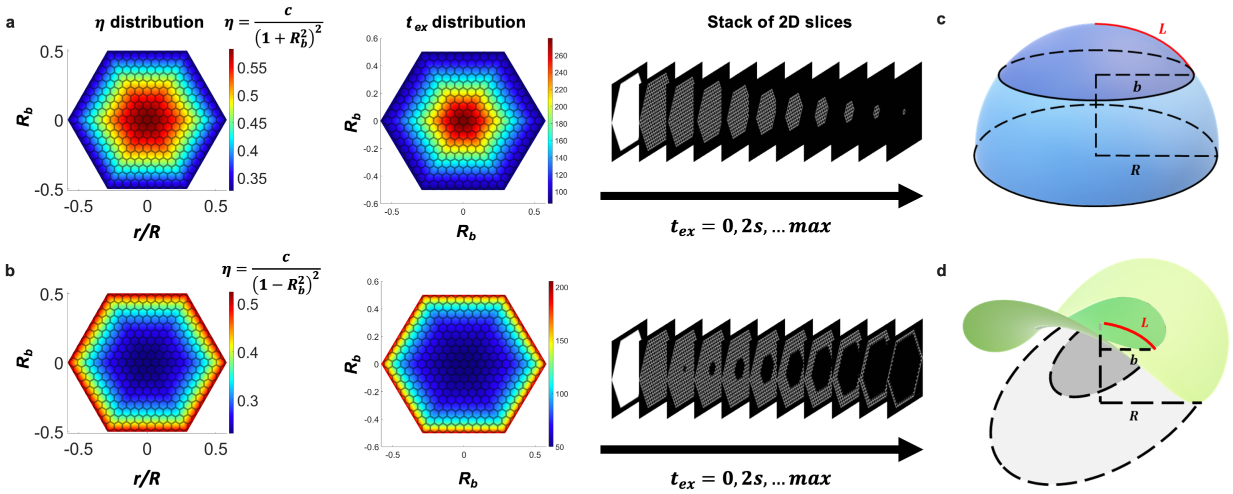

Supplementary Fig. 22. Design principles to encode gradients, facilitating the transition from 2D HC-120 hydrogel films to non-Euclidean 3D shapes. a, Encoding a spherical cap using the growth function $\eta = A_{35°C}/A_0 = \frac{c}{(1+R_b^2)^2}$. **b,** Encoding a hyperbolic saddle using the growth function $\eta = A_{35°C}/A_0 = \frac{c}{(1-R_b^2)^2}$, where c is constant, $R_b = b/R$ is the relative radius of each hexagon circle, and b represents the variable radius [25]. The η distribution reveals different trends in the growth function from the perimeter to the center for a spherical cap and a hyperbolic saddle. 2D slices demonstrate the stack of exposure times at different locations [34].

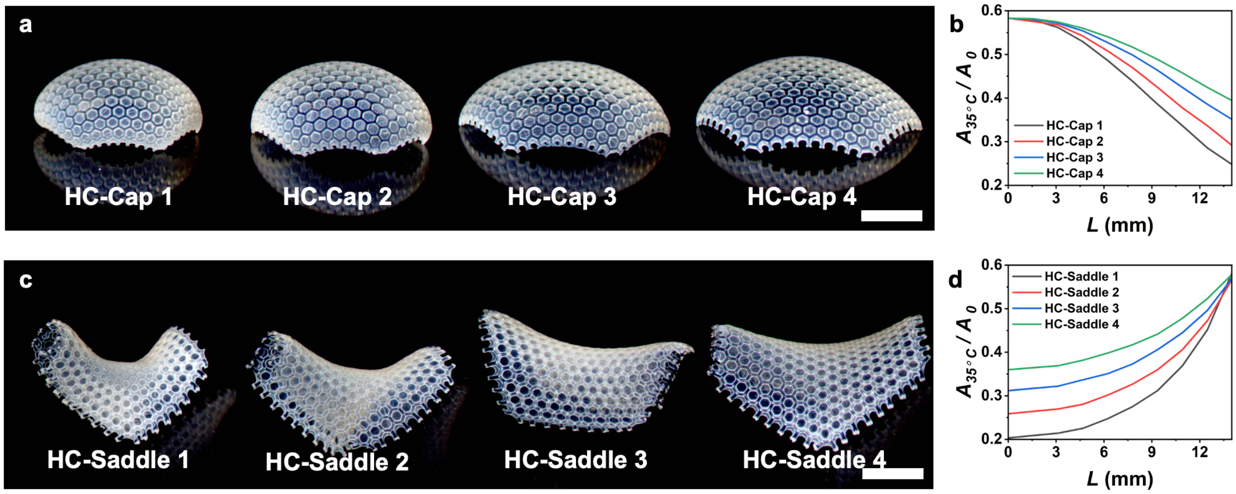

Supplementary Fig. 23. 2D-to-3D shape transformation under physiological conditions (35 °C). a,b, Precise control over the curvatures and angles in 3D caps (**a**) alongside the corresponding growth functions ($\eta = A_{35°C}/A_0$) dictating the shape transformations (**b**). **c,d,** Meticulous shaping of 3D hyperbolic saddles (**c**) with their respective growth functions guiding the shape transformations (**d**). The *L* in (**b** and **d**) represents the variable arc length on the tunable surface of the shape morphed 3D structures. The NIPAm monomer was chosen to lower the T_c of our hydrogel, enabling shape transformations near a physiological temperature (*e.g.*, 35 °C). Scale bars, 5 mm.

Supplementary Tables

Supplementary Table 1. Variations in basic parameters of hydrogel samples in reciprocal space during stretching.

	L'_a/L_a	L'_b/L_b	Poison's ratio
**HC-50**	0.826	0.940	-
**HC-90**	0.767	1.133	+
**HC-131**	0.814	1.104	+
**HC-160**	0.870	1.015	+

Notes: L_a and L_b represent the initial parameters of the hydrogel samples in the deformed state, while L'_a and L'_b represent the parameters of the hydrogels in the deformed state before failure.

Supplementary Table 2. Key parameters used in the finite element analysis (FEA) simulation.

Sample for FEM simulation	Parameter	Region	Value
HC-50 within 20s film	Elastic modulus (kPa)	Pattern domain	635
		Film domain	35
	L (mm)		2.211
	W (mm)		1.105
	t (mm)		0.32
HC-90 within 20s film	Elastic modulus (kPa)	Pattern domain	635
		Film domain	35
	L (mm)		2.25
	W (mm)		0.975
	t (mm)		0.38
HC-50 within 40s film	Elastic modulus (kPa)	Pattern domain	635
		Film domain	180
	L (mm)		2.8
	W (mm)		1.4
	t (mm)		0.35
HC-90 within 40s film	Elastic modulus (kPa)	Pattern domain	635
		Film domain	180
	L (mm)		2.3
	W (mm)		1
	t (mm)		0.38
HC-120 within 20s film	Elastic modulus (kPa)	Pattern domain	635
		Film domain	180
	L (mm)		2.55
	W (mm)		1.27
	t (mm)		0.31
HC-160-L within 20s film	Elastic modulus (kPa)	Pattern domain	635
		Film domain	180
	L (mm)		2.7
	W (mm)		1
	t (mm)		0.2

Notes: the length of the vertical wall L, the length of the inclined wall W, the wall thickness t, and the inclined angle θ are demonstrated in Supplementary Fig. 2.

Supplementary Table 3. Calculated mechanical features of specific local regions in HC-50 (20-second UV exposure for soft film domain).

**HC-50**	$\boldsymbol{\varepsilon_i}$ **(%)**	$\boldsymbol{u_i}$ $(\boldsymbol{J\,m^{-3}})$	$\boldsymbol{A_i}$ (%)	$\boldsymbol{U_i}(\boldsymbol{J\,m^{-3}})$
Subdomain A	45~57%	5.98	19.4%	1.16
Subdomain B	17~41%	3.34	40.9%	1.37
Inclined region of the patterns	2%	0.13	10.4%	0.01
Vertical region of the patterns	11%	3.84	29.3%	1.13
Total			100%	3.67

Supplementary Table 4. Calculated mechanical features of specific local regions in HC-90 (20-second UV exposure for soft film domain).

**HC-90**	$\boldsymbol{\varepsilon_i}$ **(%)**	$\boldsymbol{u_i\ (J\ m^{-3})}$	$\boldsymbol{A_i}$ **(%)**	$\boldsymbol{U_i(J\ m^{-3})}$
Subdomain A	22~42%	2.95	27.9%	0.82
Subdomain B	13~29%	1.97	37.5%	0.74
Inclined region of the patterns	3%	0.29	22.7%	0.06
Vertical region of the patterns	10%	3.18	11.9%	0.38
Total			100%	2.00

Supplementary Table 5. Calculated mechanical features of specific local regions in HC-50 (40-second UV exposure for soft film domain).

HC-50	ε_i (%)	u_i ($J\,m^{-3}$)	A_i (%)	U_i ($J\,m^{-3}$)
Subdomain A	21%	3.79	22.4%	0.85
Subdomain B	18%	2.79	46.8%	1.30
Inclined region of the patterns	5%	0.79	14.8%	0.12
Vertical region of the patterns	14%	6.22	16.0%	1.00
Total			100%	3.27

Supplementary Table 6. Calculated mechanical features of specific local regions in HC-90 (40-second UV exposure for soft film domain).

HC-90	ε_i (%)	u_i ($J\,m^{-3}$)	A_i (%)	U_i($J\,m^{-3}$)
Subdomain A	19%	3.11	22.4%	0.70
Subdomain B	12%	1.24	46.1%	0.57
Inclined region of the patterns	10%	3.18	15.0%	0.48
Vertical region of the patterns	12%	4.57	16.5%	0.75
Total			100%	2.50

Supplementary Table 7. Size-dependence of critical dimension parameters of cellular units and associated subdomains in HC-90 and HC-50 hydrogel films.

Sample	*d* (mm) @λ_y = 1.2	Err (mm)		Sample	*d* (mm) @λ_y = 1.2	Err (mm)
HC-90 subdomain A	0.388	0.02750		HC-50 subdomain A	0.300	0.01350
HC-90 subdomain B	0.254	0.01756		HC-50 subdomain B	0.259	0.01270
HC90	0.951	0.00451		HC-50	0.829	0.02146
HC-90 subdomain A	0.961	0.06421		HC-50 subdomain A	0.553	0.02563
HC-90 subdomain B	0.401	0.05751		HC-50 subdomain B	0.344	0.02354
HC90	1.610	0.04246		HC50	1.220	0.01929
HC-90 subdomain A	1.010	0.05085		HC-50 subdomain A	0.751	0.01418
HC-90 subdomain B	0.598	0.06742		HC-50 subdomain B	0.567	0.03371
HC90	2.052	0.01893		HC50	1.872	0.02802
HC-90 subdomain A	1.231	0.08031		HC-50 subdomain A	0.859	0.03995
HC-90 subdomain B	0.714	0.04557		HC-50 subdomain B	0.658	0.01587
HC90	2.323	0.02265		HC50	2.158	0.09237
