# Supplementary material for: Tailoring smart hydrogels through manipulation of heterogeneous subdomains
Source: Nat Commun. 2024 Oct 27;15:9268. doi: 10.1038/s41467-024-53552-3 (PMC11514287; doi:10.1038/s41467-024-53552-3)
Supplement: Supplementary file 2 — Description of Additional Supplementary Files [file 41467_2024_53552_MOESM2_ESM.pdf]

Description of Additional Supplementary Files

Supplementary Movie 1. In-situ observation of full-field x-strain mappings and strain profiles derived from marked linear pathways (e.g., X and Y) of HC-50 hydrogel films when stretching along the y direction.

Supplementary Movie 2. In-situ observation of full-field x-strain mappings and strain profiles derived from marked linear pathways (e.g., X and Y) of HC-90 hydrogel films when stretching along the y direction.
